# Scaffold-Mediated Microenvironmental Modulation Targeting Osteoclasts for ONFH Niche Reprogramming

**DOI:** 10.34133/research.1027

**Published:** 2025-11-26

**Authors:** Hongyu Quan, Yuwei He, Pengcheng Xiao, Zhenglin Zhu, Xiuteng Zhou, Zhong Alan Li, Yiting Lei, Hong Jiang, Wei Huang

**Affiliations:** ^1^ Department of Orthopaedic Surgery, The First Affiliated Hospital of Chongqing Medical University, Chongqing 400016, China.; ^2^ Chongqing Municipal Health Commission Key Laboratory of Musculoskeletal Regeneration and Translational Medicine, Chongqing 400016, China.; ^3^ Orthopaedic Research Laboratory of Chongqing Medical University, Chongqing 400016, China.; ^4^ Department of Biomedical Materials Science, College of Biomedical Engineering, Army Medical University (Third Military Medical University), Chongqing 400038, China.; ^5^ National Resource Center for Chinese Materia Medica, China Academy of Chinese Medical Sciences/State Key Laboratory for Quality Ensurance and Sustainable Use of Dao-di Herbs, Beijing 100700, China.; ^6^ Department of Biomedical Engineering, The Chinese University of Hong Kong, NT, Hong Kong SAR 999077, China.; ^7^ Key Laboratory of Regenerative Medicine, Ministry of Education, School of Biomedical Sciences, The Chinese University of Hong Kong, Hong Kong SAR, China.

## Abstract

Osteonecrosis of the femoral head (ONFH) is a progressive and disabling condition characterized by bone microenvironmental dysregulation, including imbalanced bone remodeling, impaired angiogenesis, and dysregulated osteoimmunity. In hip-preserving surgeries for early-stage ONFH, the most common approach is core decompression with grafting. Although widely used synthetic scaffolds, such as ceramics and metals, provide structural support or partially improve blood supply, they cannot counteract the complex pathological microenvironment of ONFH, resulting in poor long-term efficacy. To address these limitations, bioactive scaffolds incorporating functional agents have been developed to modulate pathological abnormalities and improve regenerative outcomes. This review summarizes recent advances in bioactive scaffolds for ONFH, focusing on systems functionalized with small molecules, growth factors, stem cells, exosomes, and metal ions that regulate cellular behaviors and signaling pathways in ONFH microenvironment. Emerging evidence indicates that osteoclasts exhibit heterogeneity, influencing resorptive, angiogenic, osteogenic, and immunoregulatory processes. Building on these insights, we discuss osteoclast heterogeneity and its potential relevance to ONFH, proposing that future scaffold strategies may harness osteoclast-mediated regulation to restore the osteonecrotic niche. By integrating mechanistic insights with material design, bioactive scaffolds provide a framework for targeted microenvironmental modulation and functional bone regeneration in ONFH.

## Introduction

Osteonecrosis of the femoral head (ONFH) is a progressive and debilitating orthopedic condition resulting from impaired vascular supply to the femoral head. It primarily affects adults aged 20 to 50 and, without timely intervention, progresses to subchondral bone collapse and hip joint failure, leading to severe pain and functional disability [1,2]. Globally, approximately 20 million individuals are affected by ONFH, with 8.12 million cases reported in China alone [3]. The rising incidence, driven by factors such as increased corticosteroid use, changes in lifestyle, and improved diagnostic capabilities, has made ONFH an increasingly major public health concern [1,4]. Beyond its clinical burden, ONFH also imposes heavy demands on healthcare systems, requiring long-term management, surgery, and rehabilitation. Together with its rapid progression and high disability rate, these burdens underscore the urgent need for more effective, disease-modifying treatments.

Despite substantial research efforts, the pathogenesis of ONFH remains only partially understood. Multiple mechanisms have been implicated, including disturbances in lipid metabolism, vascular insufficiency, osteocyte apoptosis, and intravascular coagulation. Among these, ischemia resulting from vascular compromise is regarded as the predominant pathological driver [5,6]. Based on its etiology, ONFH is classified into traumatic and nontraumatic forms. Traumatic ONFH typically arises from femoral neck fractures, hip dislocations, or acetabular injuries [7]. In contrast, nontraumatic ONFH is linked to various systemic risk factors, most notably long-term corticosteroid use and chronic alcohol consumption [7,8]. Other contributors include decompression sickness, autoimmune disorders, hemoglobinopathies, coagulation abnormalities, and lifestyle or environmental exposures such as smoking, obesity, radiation, and pregnancy [9]. Notably, corticosteroid-induced ONFH is the most prevalent subtype, accounting for over 50% of nontraumatic cases worldwide [10,11]. The incidence of corticosteroid-associated ONFH spiked following the severe acute respiratory syndrome outbreak, reaching up to 24% [12,13]. During the COVID-19 pandemic, corticosteroids were used in nearly one-fifth of patients in both China and the United States, raising concerns about a new surge in postpandemic ONFH cases [14,15].

The pathological progression of ONFH is marked by osteocyte apoptosis, trabecular disintegration, and extensive degradation of the bone matrix, all of which substantially elevate the risk of femoral head collapse [16]. Early diagnosis and timely intervention are therefore critical. Clinical studies report that up to 94% of untreated patients experience femoral head collapse or develop secondary osteoarthritis within 5 years, ultimately requiring total hip arthroplasty [17]. Despite this high risk, no standardized treatment protocol exists for early-stage, precollapse ONFH. Nonsurgical therapies such as medications and physiotherapy may ease symptoms or slow progression, but strong clinical evidence supporting their long-term efficacy remains limited [7,18]. As a result, surgical interventions are commonly employed in early-stage ONFH, with core decompression combined with grafting being the most widely used strategy [2,19]. A variety of grafting materials have been explored for ONFH treatment, including autografts, allografts, xenografts, and synthetic scaffolds. However, autografts are limited by donor site morbidity, while allografts and xenografts face shortages of donor sources and risks of immune rejection and poor host integration. To overcome these limitations, synthetic scaffolds such as bioceramics, polymers, metals, and composites have been developed as alternative solutions and have shown potential to enhance bone structure and support angiogenesis when combined with core decompression [20]. However, their regenerative performance remains unsatisfactory, as they fail to address ONFH’s complex pathological microenvironment, leading to poor long-term outcomes [21].

The progression of ONFH is increasingly understood to result from complex microenvironmental dysregulation. Dysfunction in local cellular populations triggers a cascade of pathological events, including imbalanced bone remodeling, impaired angiogenic function, and dysregulated osteoimmunity [22]. These abnormalities not only drive disease progression but also severely impair the regenerative potential of conventional scaffolds. Consequently, therapeutic strategies have shifted from purely mechanical reconstruction toward microenvironmental modulation. Recent efforts focus on the development of bioactive scaffolds that incorporate functional agents such as small-molecule compounds, growth factors, stem cells, exosomes, and metal ions [2]. By precisely regulating cellular behavior and signaling pathways, these scaffolds aim to restore microenvironmental homeostasis and thereby promote vascularized bone regeneration in ONFH. Although numerous studies have explored individual aspects of these processes, existing findings remain fragmented and not yet fully integrated, particularly in clarifying how distinct microenvironmental factors collectively influence repair outcomes.

**Fig. 1.**
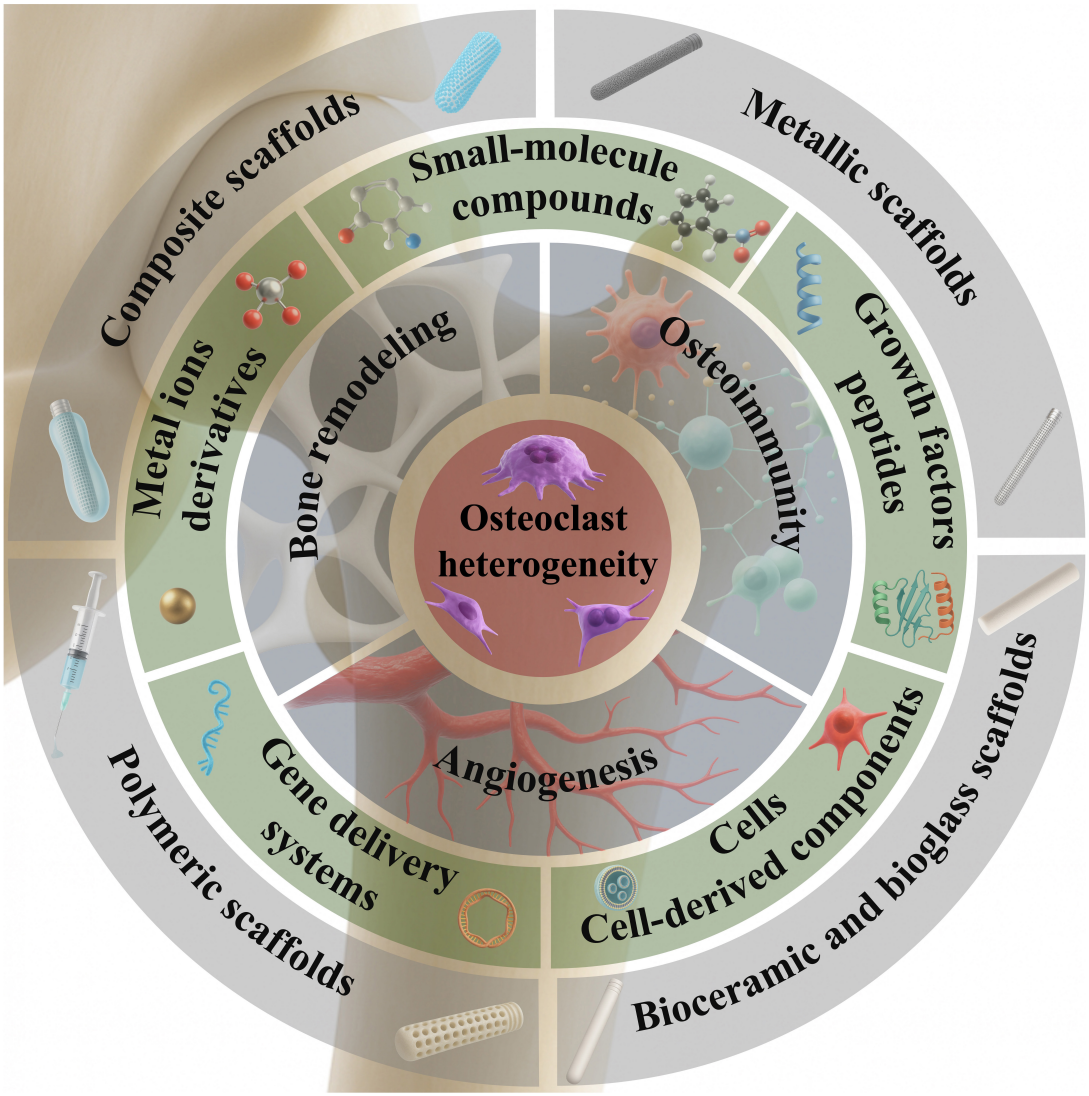
Schematic illustration of bioactive scaffold strategies targeting microenvironmental dysregulation in ONFH treatment. ONFH is characterized by disrupted bone remodeling, impaired angiogenesis, and osteoimmune imbalance. Bioactive scaffolds incorporating functional components are designed to regulate these pathological processes and promote vascularized bone regeneration. Osteoclast heterogeneity underlies the coordination of these interrelated events, offering a conceptual framework for scaffold-based regulation of the osteonecrotic microenvironment.

Given these fragmented insights, a multidisciplinary synthesis is needed to clarify how microenvironment-targeted scaffolds can advance ONFH therapy. This review begins by outlining the pathophysiological mechanisms of ONFH, with particular emphasis on microenvironmental dysregulation. We then summarize recent progress in the development of bioactive scaffolds designed to target key pathological processes, including abnormal bone remodeling, impaired angiogenesis, and osteoimmune imbalance (Fig. 1). In addition, we highlight emerging evidence of osteoclast heterogeneity, as osteoclasts regulate not only bone resorption but also angiogenesis, osteogenesis, and immune modulation within the local microenvironment. Although evidence in ONFH remains limited, findings from broader bone biology research suggest that osteoclast heterogeneity plays an important role in maintaining microenvironmental homeostasis. Building on these insights, we discuss the potential relevance of osteoclast heterogeneity to ONFH and propose that targeting osteoclast-mediated regulation could represent a promising direction for future scaffold design and microenvironmental restoration.

## Pathological Disruption of the Bone Microenvironment in ONFH

The bone microenvironment is a complex and dynamic system composed of cellular components (osteoblasts, osteocytes, mesenchymal stem cells [MSCs], vascular endothelial cells, immune cells, and neural cells) and noncellular components (extracellular matrix, soluble signaling molecules, and vascular networks) [23]. Under physiological conditions, these elements function synergistically to maintain bone metabolic homeostasis, thereby preserving skeletal structure and function. In ONFH, however, this balance is disrupted by cellular dysfunction, leading to progressive structural deterioration of the femoral head. The pathological hallmarks of this microenvironmental dysregulation include imbalanced bone remodeling, impaired angiogenesis, and dysregulated osteoimmunity, all of which collectively impair regenerative capacity and accelerate disease progression [22,23].

### Imbalanced bone remodeling

Bone homeostasis depends on the tightly regulated balance between osteoblast-driven bone formation and osteoclast-mediated bone resorption. Under normal physiological conditions, osteoblasts drive bone formation by synthesizing collagen and depositing mineralized matrix, whereas osteoclasts mediate bone resorption through acidification and proteolytic degradation, thereby maintaining structural integrity and metabolic equilibrium [24]. Osteocytes, the most abundant bone cell type, act as mechanosensors and coordinate osteoblast and osteoclast functions through secreted factors such as sclerostin [25]. They also contribute to bone matrix mineralization and secrete vasoactive molecules to support vascular function, facilitating bone–vascular coupling [25].

In ONFH, this balance is severely disrupted, triggering destructive remodeling processes [26]. This disruption is primarily initiated by ischemia- and steroid-induced oxidative stress, which alters osteoblast–osteoclast signaling and shifts bone turnover toward excessive resorption. In necrotic areas, osteoblast number and activity decline markedly. This decline coincides with reduced expression of osteogenic regulators such as runt-related transcription factor 2 and bone morphogenetic protein (BMP). The resulting loss of collagen synthesis and mineral deposition weakens trabecular structure and increases susceptibility to microcracks [27].

At the same time, hyperactive osteoclasts secrete excessive acidic by-products. This localized acidification dissolves mineralized bone, activates matrix metalloproteinase (MMP), and further degrades the extracellular matrix [26]. Acidic pH also enhances osteoclastogenesis via activation of proton-sensing receptors and receptor activator of nuclear factor-κB ligand (RANKL) signaling, creating a feed-forward loop that amplifies bone resorption. Conversely, inhibition of osteoclast activity, which buffers extracellular acidification and limits MMP activity, has been shown to mitigate trabecular degradation, underscoring the causal role of osteoclasts in ONFH-related bone loss [2].

Osteocyte apoptosis and functional impairment diminish regulatory feedback to both osteoblasts and osteoclasts, exacerbating matrix degradation and mineralization disorders. Loss of osteocyte-derived RANKL and sclerostin disrupts bone formation–resorption coupling and spatial coordination, leading to porous trabeculae and microcavity development [28]. Conversely, preservation of osteocyte viability has been shown to maintain remodeling coherence and prevent localized stress concentration [29].

These alterations ultimately destabilize bone architecture by disrupting the mechanical–biological feedback that maintains trabecular integrity. The resulting stress concentration in weakened trabeculae further accelerates osteocyte apoptosis and osteoclast overactivation, perpetuating a vicious cycle that culminates in femoral head collapse [30].

### Impaired angiogenic function

The vasculature within the bone microenvironment plays a central role in maintaining skeletal homeostasis by delivering oxygen and nutrients, removing metabolic waste, and coordinating angiogenesis with osteogenesis through paracrine signaling [31]. Under physiological conditions, angiogenesis precedes and supports bone formation, ensuring a tightly regulated coupling between vascular and skeletal systems. Endothelial cells secrete vasoactive molecules such as vascular endothelial growth factor (VEGF) and platelet-derived growth factor (PDGF). These factors promote endothelial proliferation and migration while also stimulating osteoblast differentiation and matrix mineralization [32]. Newly formed blood vessels integrate into surrounding bone tissue, ensuring sufficient perfusion to sustain metabolic activity and support continuous bone remodeling.

In ONFH, angiogenic function is markedly impaired, resulting in local ischemia and progressive degeneration of vascular networks within necrotic regions [33]. Ischemia-induced endothelial dysfunction leads to decreased VEGF expression and reduced endothelial progenitor recruitment, impairing the formation of type H vessels that normally couple angiogenesis and osteogenesis [34]. Neovascularization is largely restricted to the periphery, while the central necrotic core remains poorly perfused due to the formation of a sclerotic barrier [30]. This architectural obstruction not only prevents vascular ingrowth but also establishes a hypoxic and nutrient-deficient microenvironment that further suppresses osteogenic activity.

Vascular deficiency not only fails to meet the metabolic demands of newly forming bone but also decouples angiogenesis from osteogenesis, profoundly compromising osteoblast function and regenerative potential. Reduced endothelial paracrine signaling, such as diminished secretion of VEGF, nitric oxide, and angiopoietin (ANG), weakens osteoblast survival and differentiation, creating a feed-forward loop of vascular and osteogenic failure. Moreover, reduced angiogenesis induces hypoxia, leading to excessive accumulation of reactive oxygen species (ROS) [35]. ROS-mediated oxidative stress induces mitochondrial dysfunction in osteoprogenitors and mature osteoblasts while simultaneously stimulating RANKL expression and osteoclast overactivation, thereby intensifying bone resorption [35].

Together, these findings identify impaired angiogenesis as a key causal link between vascular collapse and bone degeneration. It integrates metabolic stress, oxidative injury, and disordered remodeling into a unified pathogenic process. Conversely, restoration of vascular perfusion or enhancement of VEGF-mediated signaling has been shown to reestablish angiogenic–osteogenic coupling, attenuate oxidative stress, and promote structural repair in ONFH [33]. This evidence supports the view that vascular dysfunction is not merely a secondary effect of necrosis but a primary driver of microenvironmental collapse and bone failure.

### Dysregulated osteoimmunity

Osteoimmunity plays a pivotal role in regulating bone metabolism by integrating innate and adaptive immune mechanisms with skeletal responses [36,37]. Under physiological conditions, a dynamic equilibrium is maintained between components of the innate immune system, such as pro-inflammatory M1 and anti-inflammatory M2 macrophages, and those of the adaptive immune system, including T and B lymphocytes. M2 macrophages secrete anti-inflammatory cytokines such as interleukin-10 (IL-10) and transforming growth factor-β (TGF-β), which enhance osteoblast differentiation and matrix mineralization [38]. They also release pro-angiogenic factors such as VEGF and PDGF, thereby coupling immunomodulation with vascular repair. Meanwhile, T cells regulate osteoblast and osteoclast functions through cytokine secretion, and B cells influence matrix metabolism via antibody and signaling molecule production [36]. This coordinated immune–skeletal–vascular network ensures balanced remodeling and stable bone homeostasis.

In ONFH, this immune equilibrium collapses, giving rise to persistent inflammation and defective tissue regeneration. Ischemia and necrosis release damage-associated molecular patterns, which activate pattern recognition receptors such as toll-like receptors on macrophages, driving polarization toward the M1 phenotype [36]. These macrophages secrete pro-inflammatory cytokines, including IL-1β, tumor necrosis factor-α (TNF-α), and IL-6 [39].

Through activation of nuclear factor-κB (NF-κB) and Janus kinase/signal transducer and activator of transcription pathways, these cytokines suppress osteoblast differentiation and up-regulate RANKL expression in stromal cells, thereby enhancing osteoclastogenesis and bone resorption [40]. Concurrently, inflammatory mediators impair endothelial integrity and suppress angiogenic signaling by reducing VEGF and ANG expression, leading to endothelial apoptosis and vascular rarefaction. The resulting hypoxic stress further reinforces M1 polarization through hypoxia-inducible factor 1α signaling, establishing a vicious feedback loop that links immune activation, vascular regression, and bone loss.

Persistent inflammatory activation also reshapes adaptive immunity. Prolonged cytokine stimulation skews T-cell differentiation toward Th1 and Th17 subsets [39,41]. Th1 cells release interferon-gamma (IFN-γ), while Th17 cells secrete IL-17, both of which strongly inhibit the osteogenic differentiation of MSCs and disrupt osteoblast–osteoclast coupling [36]. Concurrently, reduced regulatory T-cell (Treg) activity weakens anti-inflammatory control, allowing excessive cytokine amplification and prolonged tissue injury [39]. Dysregulated B-cell activity further contributes to matrix degradation by producing inflammatory mediators and autoantibodies that disrupt bone microarchitecture [39]. These interconnected processes create a causal cascade wherein chronic immune activation suppresses osteogenesis, exacerbates bone resorption, and perpetuates microenvironmental instability.

Conversely, modulation of the immune microenvironment through restoring the balance between M1 and M2 macrophage polarization and enhancing Treg function has been shown to attenuate inflammation and reestablish osteoimmune homeostasis [36]. These changes help restore angiogenic–osteogenic signaling and promote bone repair in ONFH. These findings underscore that immune dysregulation is not merely a by-product of necrosis but a key causal driver that links inflammation, impaired regeneration, and structural collapse of the femoral head.

Taken together, bone microenvironmental dysregulation in ONFH reflects a complex interplay among imbalanced bone remodeling, impaired angiogenesis, and dysregulated osteoimmunity. These disturbances not only accelerate disease progression but also undermine the efficacy of conventional scaffolds. To overcome these challenges, research has increasingly shifted from purely mechanical reconstruction toward microenvironment-targeted strategies, laying the foundation for the development of next-generation therapeutic scaffolds.

## Current Advances in Bioactive Scaffolds for ONFH Treatment via Microenvironmental Modulation

The bone microenvironment plays a pivotal role in the onset and progression of ONFH. Conventional scaffolds that mainly provide structural support are therefore insufficient to address the underlying pathological processes. In contrast, bioactive scaffolds designed to modulate the local microenvironment have emerged as a promising approach to improve treatment outcomes. Recent advances have focused on integrating bioactive components capable of regulating bone remodeling, angiogenesis, and osteoimmune responses, thereby overcoming the limitations of conventional scaffolds [2,42]. These scaffolds are functionalized with small-molecule drugs, growth factors, cell-derived products, gene delivery vectors, and metal ions, each targeting specific microenvironmental dysregulations [2,42]. Although most of these strategies remain in the preclinical stage, they offer important insights into the therapeutic potential of microenvironment-targeted interventions and provide a foundation for future translational efforts. In this section, we review recent progress in bioactive scaffolds for ONFH therapy, categorizing them according to the incorporated bioactive agents and their roles in modulating distinct pathological pathways.

### Bioactive Scaffolds Incorporating Small-Molecule Compounds

Small-molecule compounds have emerged as a central component of bioactive scaffolds because of their well-defined chemical structures, controllable functionalization, and high physicochemical stability [43–45]. Building on the pathological insights outlined above, these compounds help modulate the disrupted bone microenvironment in ONFH. They suppress bone resorption, alleviate oxidative and inflammatory stress, and promote osteogenic and angiogenic regeneration. They can be broadly categorized into synthetic agents (e.g., bisphosphonates and catechol quinone derivatives) and natural products (e.g., icariin, notoginsenoside R1, and total flavonoids from *Dipsacus asperoides*) [2]. Among these, bisphosphonates and icariin have been most extensively studied and serve as representative examples of small-molecule-based therapeutic scaffolds for early-stage ONFH.

**Fig. 2.**
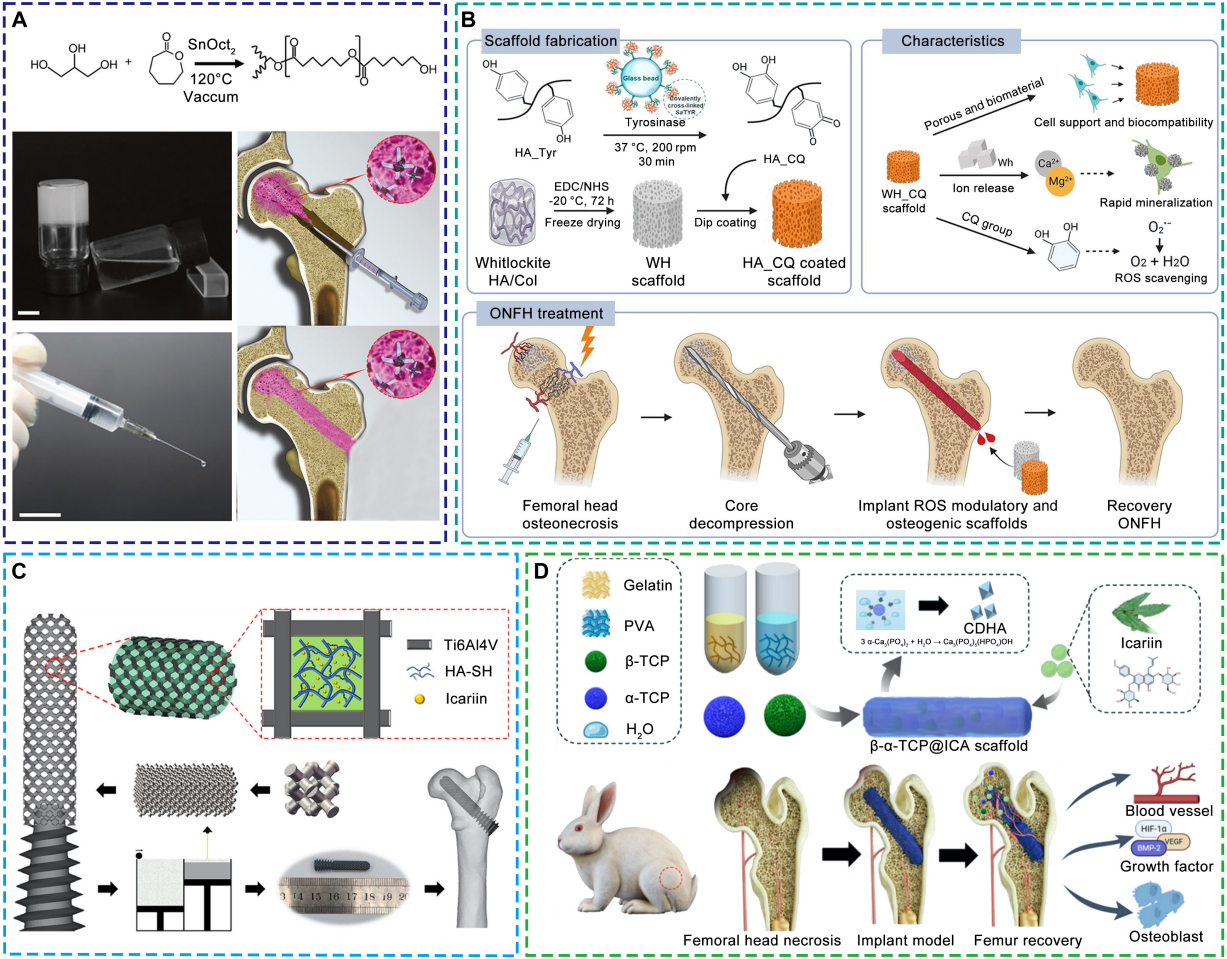
Representative bioactive scaffolds loaded with small-molecule compounds for the treatment of early ONFH. (A) Injectable glycerol-modified poly(ε-caprolactone) hydrogel loaded with zoledronic acid [48]. Licensed under a Creative Commons CC BY license. (B) Whitlockite-based scaffold coated with catechol quinone-functionalized hyaluronic acid [49]. Copyright 2023, Elsevier. (C) 3D-printed porous titanium alloy rod loaded with icariin via thiolated hyaluronic acid hydrogel [54]. Copyright 2023, Elsevier. (D) Icariin-functionalized β-TCP scaffold [55]. Copyright 2025, Elsevier.

Bisphosphonates, a classical class of antiresorptive drugs, primarily target osteoclast-mediated bone resorption by binding to hydroxyapatite (HA) and inhibiting farnesyl pyrophosphate synthase [46]. Their incorporation into scaffolds enhances local drug retention and sustains antiresorptive efficacy, as demonstrated by zoledronic acid-functionalized HA granules [47] and an injectable poly(ε-caprolactone) hydrogel loaded with zoledronic acid (Fig. 2A) [48]. Catechol quinone derivatives act through antioxidant and anti-inflammatory mechanisms that protect osteogenic and vascular cells from oxidative injury. Their incorporation into scaffolds has been shown to mitigate inflammatory responses and restore local microcirculation, exemplified by a whitlockite-based scaffold coated with catechol quinone-functionalized hyaluronic acid (Fig. 2B) [49]. Icariin, a bioactive flavonoid derived from *Epimedium brevicornum*, displays dual regulatory activity on osteogenesis and angiogenesis while concurrently suppressing bone resorption [50–52]. It promotes osteogenic differentiation and enhances endothelial proliferation and tube formation, supporting coordinated bone–vascular regeneration. Representative examples include an icariin-functionalized 3D-printed poly(lactic-co-glycolic acid) (PLGA)/tricalcium phosphate (TCP) scaffold [53], a porous titanium alloy rod coated with a thiolated hyaluronic acid hydrogel containing icariin (Fig. 2C) [54], and an icariin-functionalized β-TCP scaffold (Fig. 2D) [55].

Overall, these findings indicate that small molecule-modified scaffolds can precisely modulate pathological signaling within the necrotic microenvironment, including excessive bone resorption, oxidative stress, and inflammatory activation. Through controllable release and synergistic biological regulation, these scaffolds provide a rational strategy for restoring bone–vascular–immune homeostasis and promoting early-stage femoral head repair.

### Bioactive Scaffolds Incorporating Growth Factors and Peptides

Growth factors and bioactive peptides are widely employed in scaffold design because of their potent ability to regulate cellular signaling, promote osteogenesis, stimulate angiogenesis, and modulate osteoimmune responses [56]. Building on the pathological framework described above, these biomacromolecules help restore disrupted communication among osteogenic, vascular, and immune cells in ONFH. Commonly employed growth factors include VEGF, BMP, fibroblast growth factor (FGF), and erythropoietin [2], with VEGF and BMP being the most extensively investigated.

**Fig. 3.**
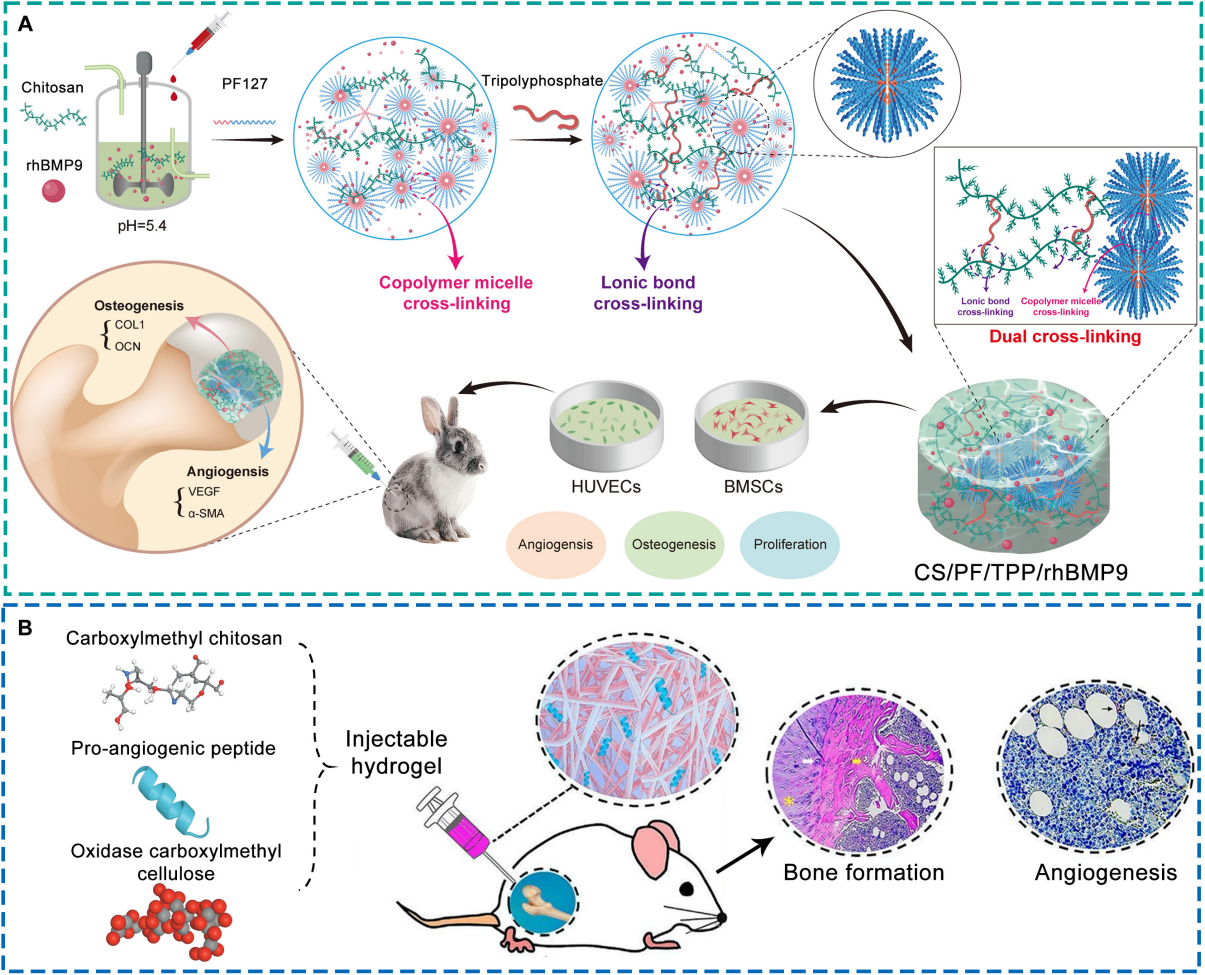
Representative bioactive scaffolds loaded with biomacromolecules and peptides for the treatment of early ONFH. (A) Injectable chitosan-based hydrogel loaded with BMP-9 [65]. Copyright 2025, Elsevier. (B) Injectable oxidized carboxymethyl cellulose-carboxymethyl chitosan hydrogel loaded with QK peptide [70]. Copyright 2024, Elsevier.

VEGF enhances angiogenesis by stimulating endothelial cell proliferation, migration, and lumen formation, thereby improving local perfusion and oxygen delivery. It also directly promotes osteogenic differentiation of MSCs, facilitating bone–vascular coupling essential for early-stage bone repair [57–60]. Controlled-release scaffolds incorporating VEGF have shown improved vascularization and bone regeneration in ONFH models. Representative examples include PLGA microspheres embedded in a PLGA-methoxy polyethylene glycol thermosensitive hydrogel [61], and gelatin microspheres delivering VEGF within a calcium phosphate scaffold [62]. BMP, among the most potent osteoinductive cytokines, drives MSC differentiation and bone matrix mineralization via calcium salt deposition [63]. Delivery of BMP through scaffolds has markedly enhanced new bone formation in necrotic regions. Examples include a gelatin-heparin-tyramine hydrogel loaded with BMP-2 [64] and a chitosan-based injectable hydrogel delivering BMP-9 (Fig. 3A) [65]. In addition, platelet-rich plasma and its lysates, enriched in PDGF, stromal cell-derived factor-1α, and TGF-β, have been used to coordinate osteogenic, angiogenic, and anti-inflammatory responses [66]. Their efficacy has been exemplified by a PLGA-HA thermosensitive hydrogel incorporating hyperactivated platelet lysates [67].

Peptides have emerged as rational alternatives to full-length growth factors, offering superior structural stability, reduced immunogenicity, and greater design flexibility. By mimicking critical bioactive domains, peptide-modified scaffolds can achieve targeted regulation of specific cell functions. Peptide-functionalized scaffolds have shown promising results in early ONFH models. For instance, a TCP scaffold modified with MSC-affinity peptide D7 enhanced MSC adhesion, spreading, and proliferation, leading to improved bone regeneration due to the dual osteogenic and angiogenic roles of MSCs [68,69]. Similarly, the VEGF-mimetic QK peptide, derived from the α-helical region of VEGF, has shown synergistic osteogenic and angiogenic activity. When incorporated into an injectable hydrogel based on oxidized carboxymethyl cellulose and carboxymethyl chitosan, QK peptide promoted MSC osteogenesis, extracellular matrix mineralization, and endothelial cell function. These effects collectively accelerated angiogenesis and bone regeneration in ONFH models (Fig. 3B) [70].

Together, these findings demonstrate that growth factor- and peptide-based scaffolds enable precise and dynamic restoration of the cellular signaling networks disrupted in ONFH. Through controlled release and multitarget regulation of osteogenic, angiogenic, and immune pathways, these biomacromolecular scaffolds provide a robust strategy for reestablishing microenvironmental homeostasis and promoting functional bone regeneration.

### Bioactive Scaffolds Incorporating Cells and Cell-Derived Components

Cells and their derivatives, particularly stem cells and exosomes, represent an advanced class of bioactive components capable of dynamically restoring the dysregulated bone microenvironment in ONFH. By integrating cellular signaling, paracrine modulation, and matrix remodeling, these systems directly address the cascade of pathological events described above, including impaired osteogenesis, vascular deficiency, and inflammatory imbalance [71]. Among them, MSCs and endothelial progenitor cells (EPCs) have demonstrated therapeutic potential in the early treatment of ONFH.

**Fig. 4.**
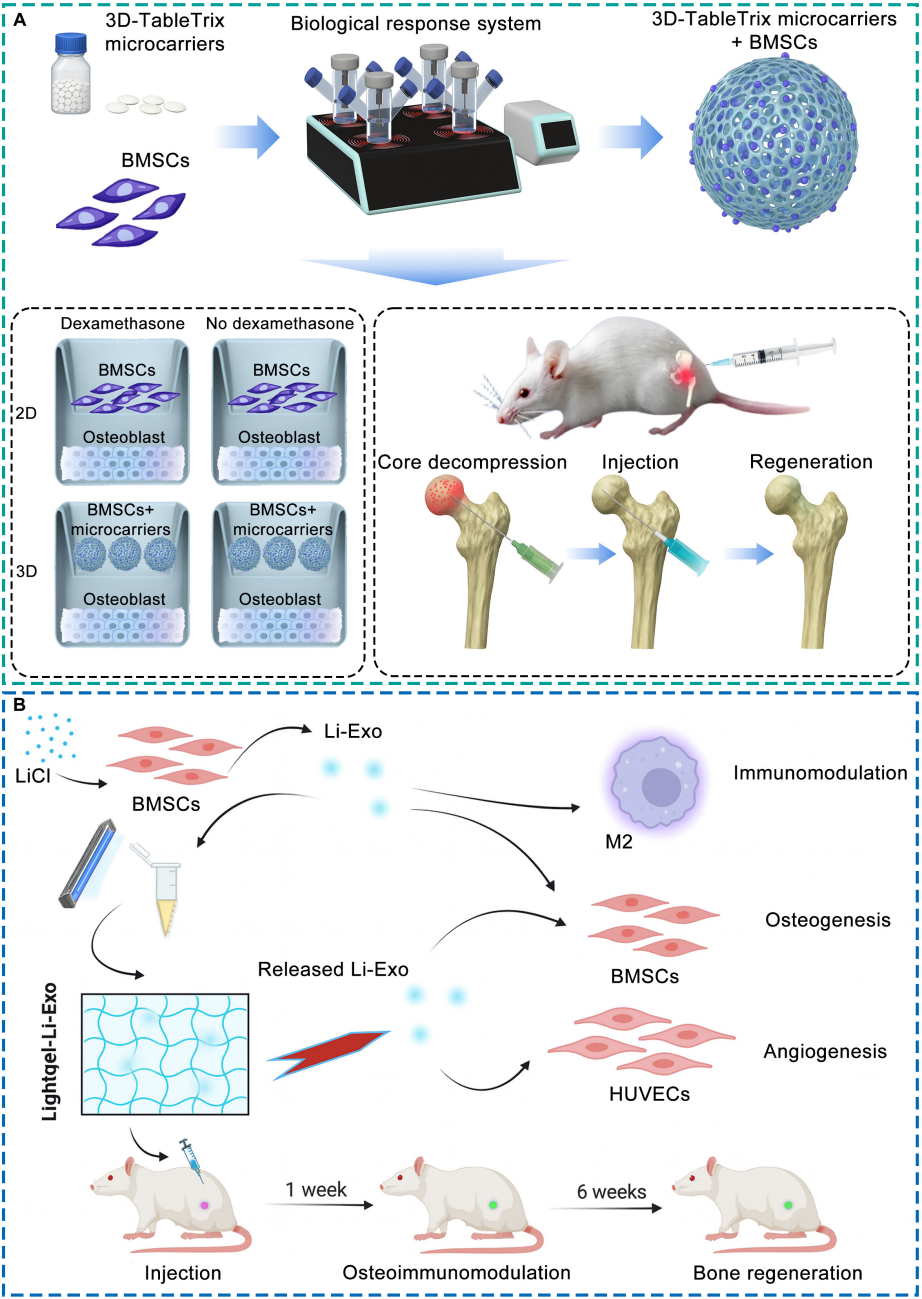
Representative bioactive scaffolds loaded with cells and cell-derived components for the treatment of early ONFH. (A) 3D gelatin micro-scaffold loaded with MSCs [80]. Copyright 2025, Elsevier. (B) Injectable methacrylated collagen hydrogel loaded with lithium-stimulated MSC-derived exosomes [82]. Copyright 2023, American Chemical Society.

MSCs modulate the bone microenvironment through both multilineage differentiation and paracrine signaling. They secrete angiogenic and immunomodulatory factors such as VEGF, FGF, IL-10, and TGF-β, which together stimulate angiogenesis, promote osteogenesis, suppress inflammation, and modulate bone resorption [72–74]. Furthermore, MSC-derived osteoprotegerin (OPG) antagonizes RANKL-mediated osteoclastogenesis, helping to reestablish the coupling between bone formation and resorption [75,76]. EPCs exert similar regulatory functions, primarily by differentiating into endothelial cells and secreting pro-angiogenic cytokines such as VEGF, thereby promoting neovascularization and improving local perfusion [77]. The resulting increase in oxygen and nutrient supply supports osteoblast survival and function, while EPC-derived anti-inflammatory mediators alleviate ischemia-induced immune activation, collectively contributing to vascular–osteogenic recovery [60,77]. Scaffolds incorporating MSCs or EPCs, either individually or in combination, have consistently demonstrated enhanced vascularization, osteogenesis, and structural regeneration in preclinical ONFH models. Representative examples include a chitosan/alginate scaffold coloaded with MSCs and EPCs [78], a gelatin-based injectable hydrogel encapsulating MSCs [79], and a 3D gelatin micro-scaffold seeded with MSCs (Fig. 4A) [80].

Compared with direct cell transplantation, exosome-based approaches offer improved stability, lower immunogenicity, and greater potential for clinical translation. Exosomes, nano-sized extracellular vesicles enriched in proteins, nucleic acids, and lipids, mimic the paracrine effects of their parent cells by regulating osteogenic, angiogenic, and immune signaling pathways [81]. They maintain the therapeutic bioactivity of MSCs and EPCs while circumventing issues of cell survival and immune compatibility. Incorporation of exosomes into scaffolds enables sustained release and spatially targeted delivery within necrotic bone regions, allowing continuous modulation of the local microenvironment. For example, lithium-preconditioned MSC-derived exosomes embedded in a methacrylated collagen hydrogel have been shown to enhance angiogenesis, suppress inflammation, and promote bone regeneration in ONFH models (Fig. 4B) [82].

Overall, cell- and exosome-based bioactive scaffolds restore cellular homeostasis by integrating osteogenic, angiogenic, and immunomodulatory functions. By activating multiple regeneration-related pathways, these systems repair structural defects and reprogram the pathological microenvironment in ONFH. Exosome-loaded scaffolds, in particular, offer a clinically adaptable platform combining safety, scalability, and sustained bioactivity, making them a promising next-generation therapeutic strategy for microenvironment-targeted bone regeneration.

### Bioactive Scaffolds Incorporating Gene Delivery Systems

Gene therapy represents a frontier strategy in bone tissue engineering. It enables precise regulation of cellular behavior and signaling pathways involved in bone remodeling, angiogenesis, and osteoimmune modulation [71]. By directly manipulating gene expression, these systems overcome major limitations of conventional bioactive agents, such as the short half-life of proteins, instability of small molecules, and limited viability of transplanted cells [83,84]. Common gene intervention tools include miRNAs, siRNAs, circRNAs, and the CRISPR system, which act by silencing pathological targets or activating regenerative pathways [83]. Their therapeutic efficacy relies on the development of efficient vectors such as nanostructured materials, synthetic polymers, and DNA-based frameworks, which ensure controlled, localized, and sustained gene delivery within the bone microenvironment.

**Fig. 5.**
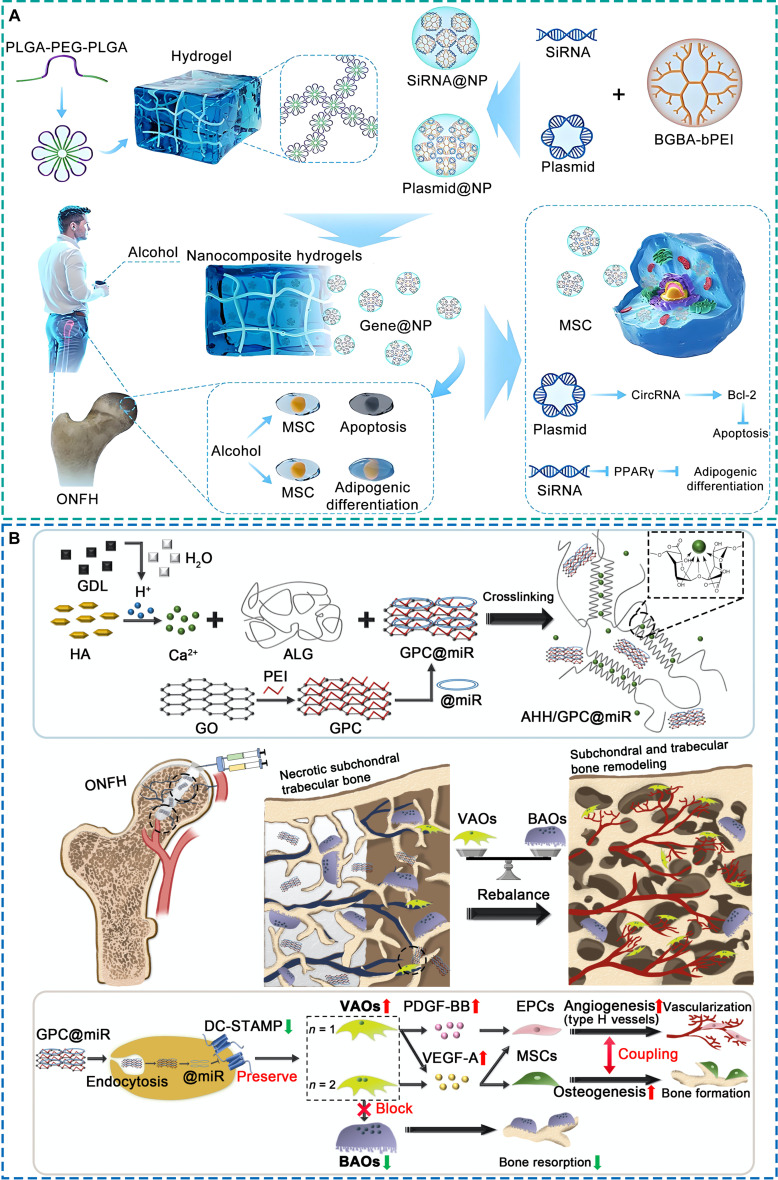
Representative bioactive scaffolds loaded with gene carriers for the treatment of early ONFH. (A) Injectable thermosensitive PLGA-PEG hydrogel loaded with a nano-gene carrier delivering circRNA-3503 plasmid and PPARγ siRNA [86]. Licensed under a Creative Commons CC BY license. (B) Injectable AHH loaded with GPC@miR [87]. Copyright 2025, Elsevier.

Although still in the early stages of application, gene-activated scaffolds have shown encouraging potential in reprogramming the ONFH microenvironment. For instance, Li et al. [85] developed an injectable lithium-heparin hydrogel incorporating a tetrahedral DNA nanostructure loaded with miR-335-5p. This system activated wingless-type MMTV integration site family (WNT) signaling pathway and suppressed Dickkopf-1 expression, resulting in enhanced osteogenesis and angiogenesis, and markedly improved tissue repair in early-stage ONFH models. In another study, Fu et al. [86] designed a dual-functional gene delivery system composed of polyethyleneimine nanocarriers modified with biguanide-4-aminobenzoic acid, coloaded with circRNA-3503 plasmids and peroxisome proliferator-activated receptor gamma (PPARγ) siRNA (Fig. 5A). Delivered via a thermosensitive PLGA-PEG hydrogel, the system down-regulated PPARγ to reduce MSC adipogenesis while up-regulating B-cell lymphoma 2 to suppress apoptosis, ultimately promoting bone regeneration and femoral head repair. More recently, our team developed an alginate/HA hydrogel (AHH) embedded with graphene oxide-based miR-7b nanocarriers (GPC@miR) for ONFH treatment (Fig. 5B) [87]. Controlled release of GPC@miR up-regulated miR-7b expression, which inhibited dendritic cell-specific transmembrane protein-mediated osteoclast fusion. This modulation restrained excessive bone resorption while preserving osteoclast populations essential for vascular and osteogenic regulation. This modulation of osteoclast function restored microenvironmental balance, improved local vascularization, and promoted bone regeneration in ONFH rat models.

Overall, gene-activated bioactive scaffolds provide a precise and multifactorial therapeutic approach to ONFH by modulating molecular networks rather than individual cellular processes. Continued advances in gene editing and delivery technologies are expected to further enhance their clinical applicability, allowing fine-tuned spatiotemporal control over bone and vascular regeneration. However, several challenges remain before clinical translation can be achieved. The safety and stability of gene vectors, as well as the off-target effects of genetic modulation, require careful evaluation to avoid unintended alterations in nonskeletal tissues. Achieving cell-type-specific targeting is therefore essential, as ectopic gene activation in vascular or immune cells could trigger unpredictable inflammatory or metabolic responses. Addressing these concerns through rational vector design, localized delivery, and temporal control will be critical to ensuring the therapeutic precision and biosafety of next-generation gene-activated scaffolds. In this context, the integration of molecular biology with materials engineering holds great promise for achieving programmable microenvironmental regulation and durable functional repair.

### Bioactive Scaffolds Incorporating Metal Ions and Their Derivatives

Metal ions and their derivatives have emerged as versatile modulators in bone tissue engineering, owing to their intrinsic antioxidant, osteogenic, angiogenic, and immunoregulatory properties [2,71,88]. When rationally incorporated into scaffolds, these ions can activate multiple signaling pathways, modulate cellular functions, and restore microenvironmental balance, thereby offering promising therapeutic potential for ONFH repair. Among them, copper (Cu^2+^), lithium (Li^+^), and manganese (Mn^2+^) are among the most extensively investigated.

**Fig. 6.**
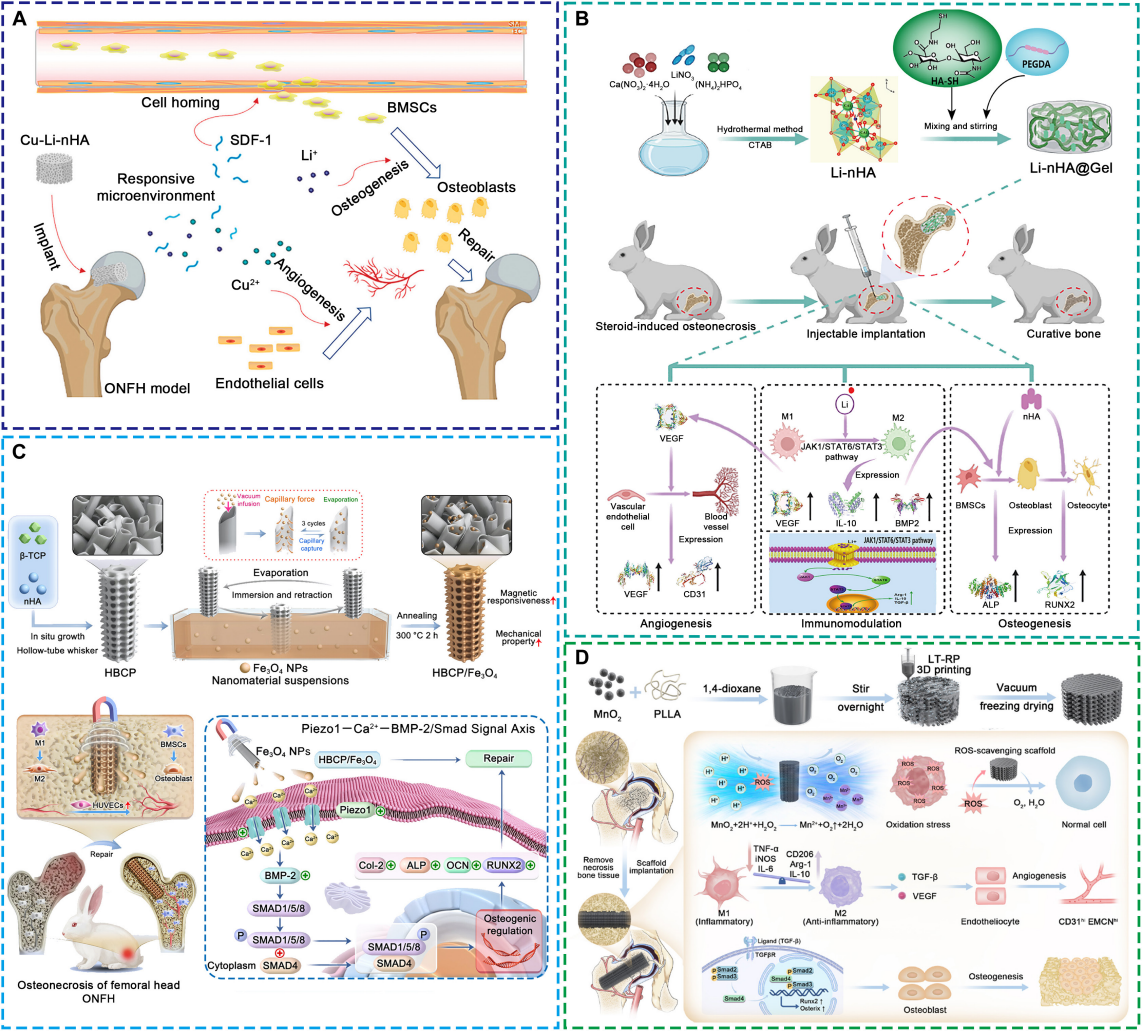
Representative bioactive scaffolds loaded with metal ions and their derivatives for the treatment of early ONFH. (A) HA scaffold loaded with copper and lithium ions [91]. Licensed under a Creative Commons CC BY license. (B) Injectable thiolated hyaluronic acid hydrogel loaded with lithium ions [92]. Copyright 2024, Elsevier. (C) Biphasic calcium phosphate scaffold incorporating Fe_3_O_4_ nanoparticles [95]. Copyright 2025, American Chemical Society. (D) 3D-printed poly-L-lactic acid scaffold loaded with manganese dioxide [35]. Licensed under a Creative Commons CC BY license.

Cu^2+^ and Li^+^ exert distinct yet complementary regulatory effects on the bone microenvironment through multiple cellular mechanisms. Cu^2+^ enhances endothelial cell proliferation and migration, facilitating neovascularization and improving tissue perfusion. It also stimulates the osteogenic differentiation of MSCs, shifts macrophage polarization toward the M2 phenotype, and suppresses osteoclastogenesis [88,89]. Li^+^ primarily enhances osteoblast differentiation and mineralization, while concurrently inhibiting osteoclastogenesis and inflammatory signaling, thereby promoting coordinated bone and vascular regeneration [88]. Several studies have demonstrated that scaffolds incorporating Cu^2+^ and Li^+^ markedly improve ONFH outcomes through these multitargeted effects. For instance, Li et al. [90,91] developed an HA scaffold coloaded with Cu^2+^ and Li^+^, enabling controlled ion release to simultaneously stimulate angiogenesis and osteogenesis, accelerating femoral head repair (Fig. 6A). Similarly, Luo et al. [92] fabricated an injectable thiolated hyaluronic acid hydrogel loaded with Li^+^, which promoted macrophage M2 polarization and suppressed inflammation, while up-regulating BMP and VEGF expression to enhance bone and vessel formation in early ONFH (Fig. 6B). Beyond Cu^2+^ and Li^+^, selenide (Se^2+^) ions have attracted interest due to their potent antioxidant and anti-inflammatory activities. By reducing oxidative stress and mitigating local inflammation, Se^2+^ enhances MSC osteogenic differentiation and endothelial cell migration, thereby facilitating coupled bone and vascular regeneration [93]. Liu et al. [94] developed an injectable carboxymethyl chitosan/alginate hydrogel incorporating Se^2+^, which protected MSCs and endothelial cells from ROS-induced damage and improved their regenerative functions, leading to improved ONFH lesion repair.

In addition to ionic forms, metal oxides have drawn increasing attention for their structural stability and multifunctionality. Zhou et al. [95] developed a magnetically responsive biphasic calcium phosphate scaffold incorporating Fe_3_O_4_ nanoparticles, which, under static magnetic field stimulation, promoted osteogenesis, angiogenesis, and immunomodulation, leading to improved bone regeneration in ONFH rabbits (Fig. 6C). Yang et al. [35] designed a 3D-printed poly-L-lactic acid scaffold incorporating MnO_2_ nanoparticles for early ONFH intervention (Fig. 6D). By scavenging ROS and releasing oxygen, MnO_2_ alleviated hypoxia-induced dysfunction of vascular and osteogenic cells. The released Mn^2+^ further promoted M2 macrophage polarization, angiogenesis, and MSC osteogenesis via TGF-β/Smad signaling. Together, these effects accelerated femoral head regeneration. More recently, Xie et al. [96] developed a 3D-printed PLGA scaffold doped with MnOx nanoparticles. This scaffold effectively relieved oxidative stress and promoted both osteogenesis and angiogenesis, resulting in enhanced vascularized bone formation in a rabbit ONFH model.

Overall, metal ions and their derivatives provide a structurally stable and mechanistically defined category of bioactive scaffolds for ONFH treatment. Through controlled ion release, these materials achieve coordinated regulation of oxidative stress, angiogenesis, osteogenesis, and immune balance within the necrotic microenvironment. However, their clinical translation requires careful control of dosage, release kinetics, and systemic toxicity, as excessive ion accumulation may disrupt cellular redox balance or trigger inflammatory cascades. Future developments should focus on optimizing ion release profiles, improving spatial targeting, and integrating multi-ion synergy with intelligent delivery systems to achieve precise and safe microenvironmental modulation.

**Fig. 7.**
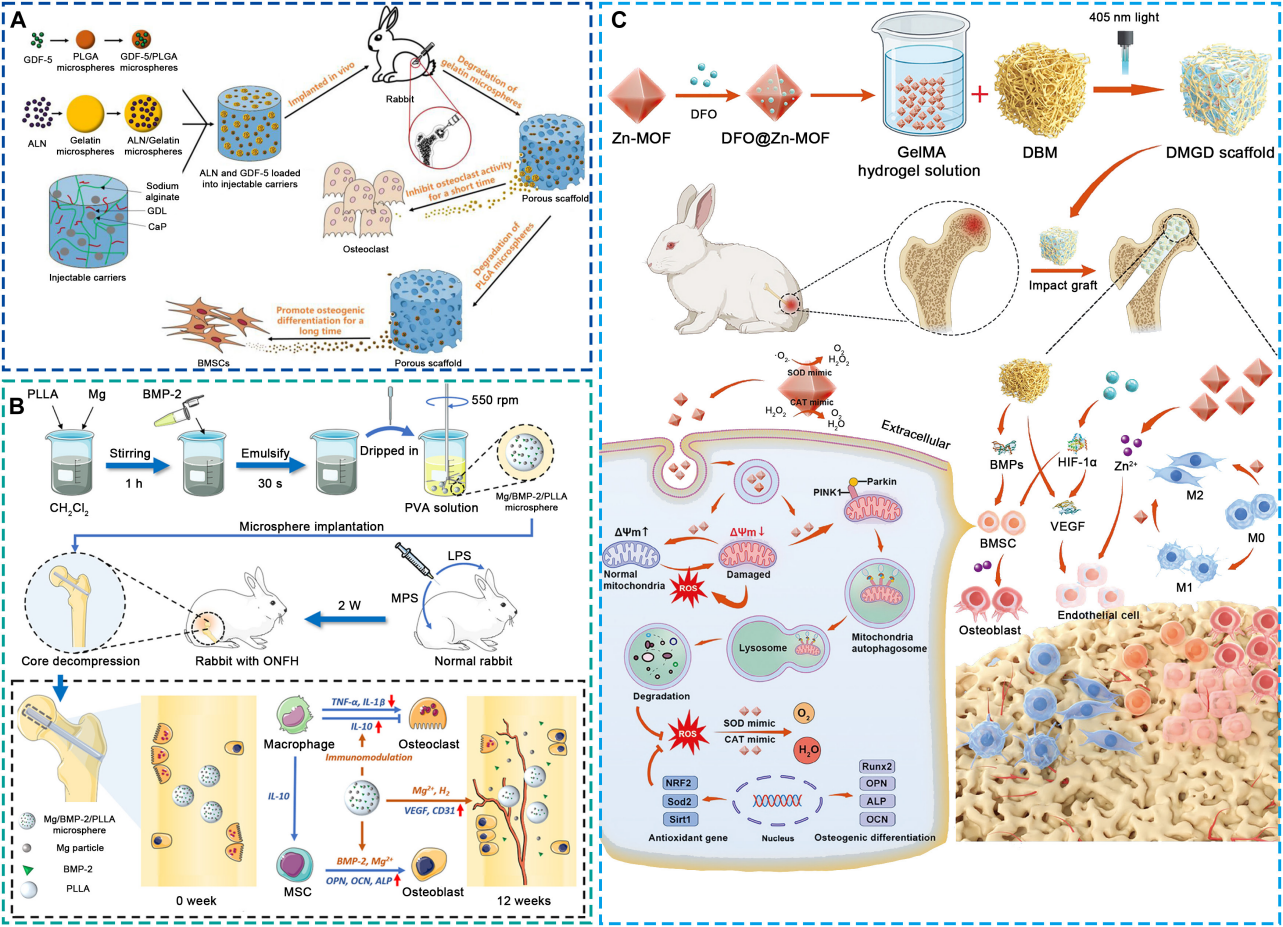
Representative bioactive scaffolds with composite loading of multiple functional substances for the treatment of early ONFH. (A) Injectable alginate-based hydrogel loaded with alendronate and growth differentiation factor-5 [97]. Copyright 2022, Elsevier. (B) Poly-L-lactic acid microspheres loaded with magnesium ions and BMP-2 [98]. Copyright 2023, Elsevier. (C) Methacrylated gelatin-demineralized bone matrix scaffold loaded with zinc ions and deferoxamine [99]. Copyright 2024, John Wiley and Sons.

Although this review categorizes bioactive scaffolds based on the type of incorporated functional agents, practical applications increasingly rely on composite systems that integrate multiple components. Such combinations aim to achieve synergistic modulation of the bone microenvironment and more comprehensively address the multifactorial pathology of ONFH. For example, Zhao et al. [97] designed an injectable alginate-based hydrogel coloaded with alendronate and growth differentiation factor-5, which concurrently inhibited bone resorption and enhanced osteogenesis, thereby promoting lesion repair (Fig. 7A). Yang et al. [98] developed poly-L-lactic acid microspheres encapsulating magnesium ions and BMP-2, demonstrating coordinated effects on angiogenesis, osteogenesis, and immune regulation (Fig. 7B). Similarly, Bai et al. [99] constructed a methylacrylated gelatin-demineralized bone matrix scaffold coloaded with zinc ions and deferoxamine (Fig. 7C). This composite scaffold mitigated oxidative stress and inflammatory responses, thereby creating a favorable microenvironment for MSC osteogenic differentiation. It also stimulated the secretion of growth factors, which enhanced both vascularization and bone formation. These examples highlight that combinatorial delivery systems can produce synergistic microenvironmental modulation across multiple pathological axes, offering a promising direction for next-generation multifunctional scaffolds.

In summary, bioactive scaffolds represent a compelling approach for early-stage ONFH treatment by enabling targeted modulation of cellular behaviors and signaling networks within the bone microenvironment. Through mechanisms such as rebalancing bone remodeling, restoring vascular function, and regulating osteoimmune interactions, these systems facilitate structural repair and functional regeneration of the necrotic femoral head. Importantly, their therapeutic performance depends not only on the identity of the incorporated agents but also on a mechanistic understanding of how these agents orchestrate cross-talk among osteogenic, angiogenic, and immune cells. A mechanism-driven design approach guided by cellular-level insights into microenvironmental regulation can optimize material selection, integration, and release kinetics. This strategy enhances therapeutic efficacy while minimizing redundancy and translational inefficiency.

In current practice, bioactive scaffolds are predominantly formulated as hydrogels or microspheres because they offer injectability, defect conformity, and precisely tunable, multiphase release. These features make them well suited to core decompression-assisted therapy in early lesions. However, limited mechanical stability constrains their stand-alone use in load-bearing zones and can shorten the window of biological benefit. To bridge this gap, hybrid composites, hierarchical architectures, and 3D-printed constructs are gaining traction. By coupling a mechanically robust framework (e.g., ceramic/polymer lattices or graded porosity struts) with bioactive layers or carriers (e.g., hydrogel shells and microsphere depots), these systems sustain localized cue delivery while maintaining structural integrity. Such designs align with surgical workflows for core decompression, extend the duration of microenvironmental modulation, and expand translational potential.

Despite encouraging preclinical progress, major challenges remain before bioactive scaffolds can be successfully translated to the clinic. Key issues include limited long-term biosafety data, variability in release kinetics, and incomplete evaluation of immune compatibility. In particular, unintended systemic diffusion or nonspecific activation of signaling pathways may lead to unpredictable inflammatory or metabolic effects. Therefore, future research should prioritize spatiotemporally controlled delivery and precise cell-type targeting that accounts for the functional heterogeneity of osteogenic, angiogenic, and osteoclastic populations within the bone microenvironment. Addressing these challenges will be crucial for transforming bioactive scaffold technology from experimental therapy to reliable clinical intervention, ultimately broadening its application beyond ONFH to other osteolytic and ischemic bone diseases.

## Osteoclast Heterogeneity and Its Regulatory Roles in Bone Microenvironmental Homeostasis

Bone homeostasis is orchestrated by a dynamic and interconnected cellular network in which osteoclasts function as pivotal regulators of skeletal remodeling and microenvironmental equilibrium. They originate from hematopoietic myeloid progenitors and form through the concerted action of macrophage colony-stimulating factor (M-CSF) and RANKL signaling [100,101]. M-CSF promotes precursor proliferation and survival, while RANKL activates NF-κB and downstream transcriptional programs that drive osteoclastogenesis and resorptive function.

Traditionally, osteoclasts have been viewed solely as bone-degrading cells functioning in opposition to osteoblasts within the remodeling cycle [102]. However, advances in single-cell transcriptomics and molecular biology have revealed that osteoclasts possess broader regulatory functions extending beyond matrix degradation. They can influence angiogenesis, osteogenesis, and immune homeostasis through the secretion of bioactive factors, presentation of membrane-bound ligands, and modulation of local signaling pathways. This recognition has shifted the understanding of osteoclasts from executors of bone resorption to coordinators within the bone–vessel–immune axis.

Recent studies in bone biology have further demonstrated that osteoclasts exhibit heterogeneity in morphology, localization, and function [103]. Resorptive osteoclasts are large multinucleated cells that form sealing zones and degrade mineralized matrix to execute bone resorption, which represents their primary function [102]. In addition to matrix degradation, these cells also contribute to angiogenic, osteogenic, and immune regulation through the release of bioactive mediators and direct cellular interactions, thereby coordinating bone resorption with broader microenvironmental processes [102]. Nonresorptive osteoclasts, typically mononuclear or nonfused, lack bone-resorptive capacity but similarly participate in angiogenic, osteogenic, and immune regulation through distinct molecular pathways and intercellular communications [103]. Although these 2 functional populations differ in morphology and activity, they do not represent separate lineages but rather exist along a dynamic continuum of differentiation, with transitions influenced by local microenvironmental cues [104]. The coexistence of such osteoclast heterogeneity reflects the multifaceted and adaptive regulatory demands of bone remodeling and microenvironmental homeostasis.

While such heterogeneity has been extensively characterized in bone biology, its pathological relevance to ONFH remains largely unexplored, with most studies still focused on resorptive osteoclast activity leading to excessive bone loss and structural collapse. Imbalance between resorptive and nonresorptive functions may underlie the impaired angiogenesis, deficient osteogenesis, and persistent inflammation characteristic of ONFH. Understanding osteoclast heterogeneity therefore provides a conceptual framework for interpreting the cellular basis of bone microenvironmental dysregulation in this disease.

The following sections summarize current knowledge on how functionally diverse osteoclasts influence bone remodeling, angiogenesis, and osteoimmunity, with emphasis on insights that may help interpret the microenvironmental disturbances characteristic of ONFH.

### Modulation of bone remodeling homeostasis by osteoclast heterogeneity

**Fig. 8.**
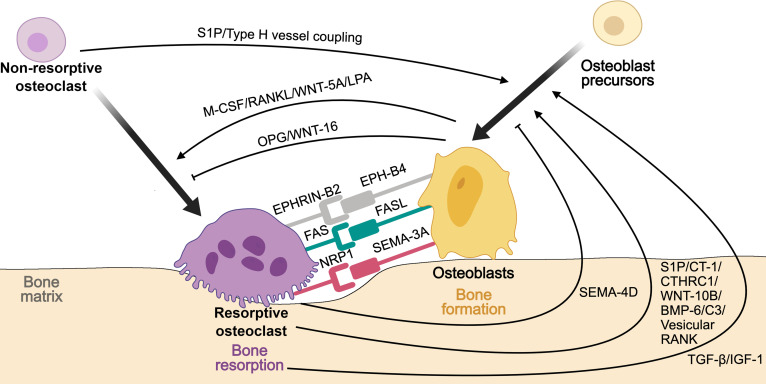
Schematic representation of osteoclast heterogeneity in the regulation of bone remodeling homeostasis. Resorptive and nonresorptive osteoclasts coordinate bone resorption and formation through multiple coupling signals with osteoblasts and their precursors. Resorptive osteoclasts release matrix-derived factors such as TGF-β and IGF-1, and secrete osteogenic mediators including BMP-6 and WNT-10B, while mediating contact-dependent signaling via EPHRIN-B2/EPHB4, FAS/FASL, and SEMA3A/NRP1 axes to stimulate osteoblast differentiation. Nonresorptive osteoclasts promote angiogenic–osteogenic coupling through the secretion of PDGF-BB and activation of S1P signaling. In turn, osteoblasts regulate osteoclast differentiation through the RANKL–RANK–OPG pathway and paracrine mediators including M-CSF, WNT-5A, and WNT-16. These reciprocal molecular interactions ensure dynamic coordination between bone resorption and formation, maintaining remodeling homeostasis.

Bone remodeling depends on the dynamic balance between bone resorption and formation, a process coordinated through reciprocal signaling between osteoclasts and osteoblasts. Although osteoclasts have long been regarded as the primary mediators of matrix degradation, accumulating evidence indicates that they also contribute to bone formation by releasing signaling molecules and regulating osteoblast activity through coupling mechanisms. These dual and partly opposing functions vary among osteoclasts with heterogeneous regulatory capacities (Fig. 8).

Resorptive osteoclasts adhere to the bone surface via integrins such as αvβ3, forming sealed resorption lacunae where H^+^-ATPase proton pumps acidify the microenvironment and enzymes such as cathepsin K degrade the organic matrix [105]. This resorption process not only removes aged bone but also releases matrix-sequestered growth factors, including TGF-β and insulin-like growth factor-1 (IGF-1), which recruit and stimulate osteoblasts, establishing a functional link between resorption and formation [100]. In physiological contexts, this coupling ensures continuous remodeling and structural renewal. However, when resorptive osteoclasts become overrepresented, as observed in ONFH, this equilibrium collapses [26]. Excessive matrix degradation and acidification damage the osteoblast niche, weaken mineralization, and accelerate trabecular fragility. Thus, the transition from balanced coupling to uncoupled remodeling reflects a shift in osteoclast functional balance rather than a mere increase in resorptive activity.

Beyond matrix-derived cues, resorptive osteoclasts can directly promote osteogenesis through soluble and contact-dependent signaling. They secrete pro-osteogenic mediators such as sphingosine-1-phosphate (S1P), WNT-10B, and BMP-6, while Ephrin-B2-Eph receptor B4 signaling mediates bidirectional communication with osteoblasts [102,106]. Moreover, apoptotic resorptive osteoclasts release extracellular vesicles and apoptotic bodies containing osteoinductive molecules that further stimulate osteoblast differentiation and matrix mineralization [107].

Nonresorptive osteoclasts, often partially differentiated or mononuclear, exhibit regulatory functions distinct from matrix degradation. Among these, preosteoclasts represent a well-characterized subset that secretes PDGF-BB and activates S1P signaling to promote H-type vessel formation and coordinate angiogenic–osteogenic coupling, thereby sustaining bone microenvironmental stability [108]. Under pathological stress such as ischemia or inflammation, osteoclastogenesis becomes skewed toward hyperactive resorptive phenotypes, leading to an overrepresentation of resorptive osteoclasts and a concomitant reduction of nonresorptive populations such as preosteoclasts [34]. This imbalance not only disrupts the temporal and spatial coordination between bone resorption and formation but also weakens angiogenic and osteogenic coupling, ultimately impairing vascular support and accelerating structural collapse in ONFH [34].

Osteoclast activity is tightly regulated by reciprocal feedback from other bone-resident cells. Osteoblasts secrete RANKL to promote osteoclastogenesis while producing OPG to restrain excessive resorptive activity, forming the RANKL–RANK–OPG axis that maintains remodeling equilibrium [100]. Perturbation of this axis, caused by altered inflammatory signaling, oxidative stress, or vascular insufficiency, results in dysregulated osteoclastogenesis and impaired coupling efficiency. As a consequence, the balance between matrix degradation and formation collapses, linking cellular heterogeneity within the osteoclast population to the macroscopic manifestations of bone necrosis and microstructural instability [26].

### Regulation of angiogenic function by osteoclast heterogeneity

**Fig. 9.**
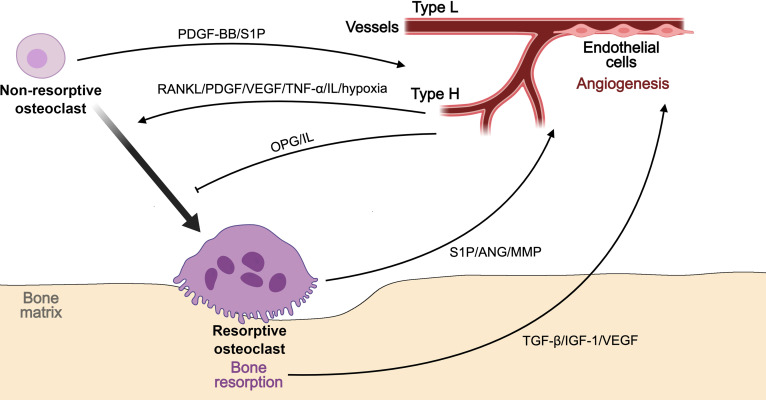
Schematic representation of osteoclast heterogeneity in the regulation of angiogenic function. Resorptive and nonresorptive osteoclasts cooperatively regulate angiogenesis through reciprocal signaling with endothelial cells. Resorptive osteoclasts promote angiogenesis indirectly by releasing matrix-derived factors such as TGF-β, IGF-1, and VEGF, and by secreting angiogenic mediators including ANG, S1P, and MMP, which activate endothelial cells and remodel the extracellular matrix. Nonresorptive osteoclasts, represented by preosteoclasts, directly enhance type H vessel formation through secretion of PDGF-BB and activation of S1P signaling. In turn, endothelial cells modulate osteoclast differentiation and activity via RANKL, VEGF, PDGF, TNF-α, and IL, thereby maintaining vascular–osteogenic coupling and microenvironmental stability. These reciprocal molecular interactions ensure dynamic coordination between bone resorption, angiogenesis, and osteogenesis, preserving vascular homeostasis within the bone microenvironment.

Beyond their classical role in bone remodeling, osteoclasts are increasingly recognized as important regulators of angiogenesis, particularly in the formation and maintenance of H-type vessels that couple vascular and osteogenic activities. These specialized vessels, closely associated with active osteogenesis, are predominantly supported by nonresorptive osteoclasts, which regulate endothelial behavior through paracrine (Fig. 9).

Resorptive osteoclasts contribute to angiogenesis not only indirectly through bone matrix degradation but also through the release of bioactive mediators. They secrete ANG to activate endothelial cells via the Plexin-B2 pathway and stabilize vessel structure and function [109]. Although direct evidence is lacking, S1P and matrix-derived factors released during bone resorption (e.g., TGF-β, IGF-1, and VEGF) are well known for their angiogenic activity, suggesting that resorptive osteoclasts may indirectly promote angiogenesis through bone remodeling [60,110,111]. Additionally, MMP released by resorptive osteoclasts degrade the matrix barrier, creating a conducive environment for angiogenesis [112].

Nonresorptive osteoclasts, including mononuclear tartrate-resistant acid phosphatase-positive preosteoclasts, exert a more direct pro-angiogenic influence. They secrete PDGF-BB, which stimulate endothelial migration and promote H-type vessel formation, thereby reinforcing the coupling between angiogenesis and osteogenesis [108,113]. These nonresorptive populations serve as key intermediaries sustaining the angiogenic niche required for bone regeneration and metabolic activity.

Reciprocal signaling between osteoclasts and endothelial cells further maintains this coordinated process. Endothelial cells secrete multiple regulatory molecules, including RANKL, VEGF, PDGF, TNF-α, and IL, which modulate osteoclastogenesis and resorptive function [114,115]. For instance, endothelial-derived VEGF and PDGF enhance RANKL-mediated signaling, coordinating osteoclast maturation and matrix degradation [116]. Conversely, impaired vascular perfusion or endothelial dysfunction alters local oxygen tension, leading to hypoxia-induced oxidative stress in osteoclasts. This condition elevates ROS levels, drives overactivation of resorptive phenotypes, establishing a feed-forward loop that links vascular regression with pathological bone destruction, as observed in osteonecrosis [16].

Altogether, these interactions indicate that angiogenic regulation is not a passive consequence of bone remodeling but an active process coordinated by osteoclast heterogeneity. Perturbations in their functional balance disrupt this vascular–osteogenic coupling, mirroring the ischemic and remodeling defects characteristic of ONFH. Recognizing the distinct yet complementary roles of these osteoclast populations provides a mechanistic bridge linking vascular insufficiency with impaired bone regeneration and underscores osteoclast heterogeneity as a crucial determinant of microenvironmental stability.

### Regulation of osteoimmunity by osteoclast heterogeneity

**Fig. 10.**
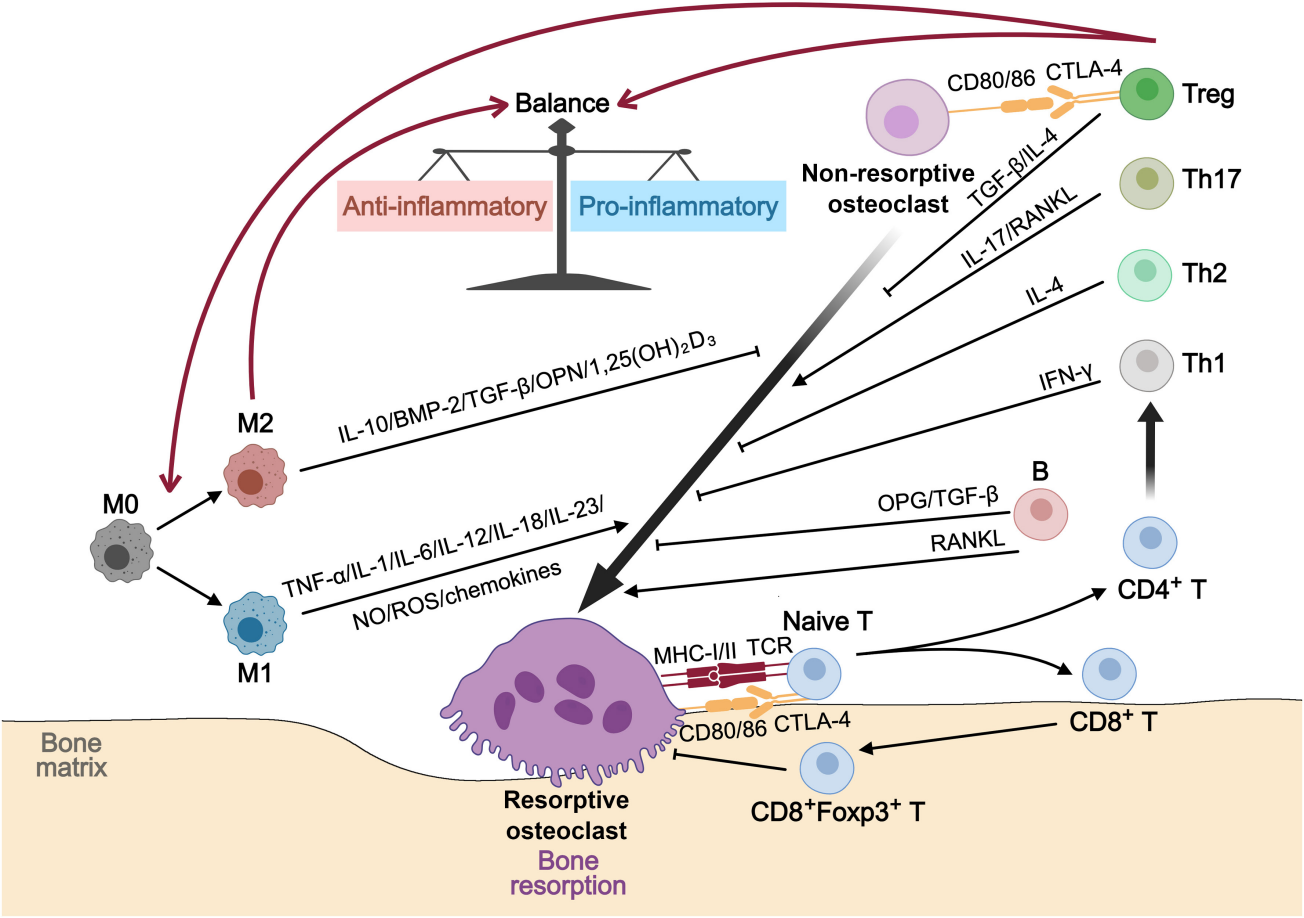
Schematic representation of osteoclast heterogeneity in the regulation of osteoimmunity. Resorptive and nonresorptive osteoclasts coordinate immune-skeletal homeostasis through bidirectional interactions with diverse immune cells. Resorptive osteoclasts act as antigen-presenting and pro-inflammatory cells: they express MHC class I/II and costimulatory molecules (CD80/CD86) to activate CD4^+^ and CD8^+^ T cells, and secrete chemokines such as CCL4 and CXCL11 that recruit and modulate immune responses. The activated Th1 and Th17 cells release cytokines including IFN-γ, TNF-α, and IL-17, which amplify inflammatory signaling and promote macrophage M1 polarization. In contrast, nonresorptive osteoclasts, particularly preosteoclasts, engage with regulatory T cells (Tregs) via CD80/CD86-CTLA-4 signaling and secrete IL-10 and TGF-β to suppress inflammatory activation and facilitate M2 macrophage polarization. Through these reciprocal interactions, immune cells in turn either promote osteoclast activation under pro-inflammatory conditions (via RANKL, TNF-α, and IL-17) or suppress excessive resorption through anti-inflammatory mediators such as IL-10 and TGF-β. These reciprocal cellular and molecular interactions integrate inflammatory and skeletal responses, maintaining immune–bone homeostasis within the microenvironment.

In addition to their roles in bone remodeling and angiogenic function, osteoclasts also serve as critical regulators of osteoimmunity. Through bidirectional interactions with both innate (e.g., macrophages) and adaptive immune cells (e.g., T and B lymphocytes), osteoclasts integrate inflammatory cues and translate them into microenvironmental responses, positioning themselves at the interface between skeletal and immune homeostasis (Fig. 10).

Among resorptive osteoclasts, certain subsets including dendritic-cell-derived osteoclasts identified under inflammatory conditions exhibit features reminiscent of antigen-presenting cells [117]. These cells express major histocompatibility complex (MHC) class I and II molecules along with costimulatory molecules such as CD80 and CD86, enabling direct interaction with T cells. For example, they secrete chemokines including C-C motif chemokine ligand 4 and C-X-C motif chemokine ligand 11 to recruit T cells, and further activate both CD4^+^ and CD8^+^ T cells via MHC and CD80/CD86 costimulatory signaling [102,118]. Once activated, these T cells secrete pro-inflammatory cytokines including TNF-α and IL-17, which enhance RANKL expression in stromal and immune cells, promote osteoclastogenesis, and amplify local inflammation. This cascade not only accelerates bone resorption but also sustains inflammatory persistence within the necrotic microenvironment [119].

Nonresorptive osteoclasts, particularly osteoclast precursors, also participate in immune regulation through cross-talk with regulatory T cells (Tregs) and macrophages. Via CD80/CD86 signaling, they modulate Treg activity, which, under physiological conditions, helps suppress effector T-cell responses and reduce local inflammation [118]. However, in pathological settings, this immunosuppressive effect may be disrupted, contributing to heightened inflammatory activity. Additionally, osteoclast–T cell interactions influence the polarization of macrophages. Cytokines secreted by activated T cells, such as IFN-γ and TNF-α, promote M1-type macrophage polarization, which amplifies pro-inflammatory responses. In contrast, anti-inflammatory cytokines released by Tregs, including IL-10 and TGF-β, support M2-type macrophage polarization and facilitate inflammation resolution [120].

Bidirectional communication between osteoclasts and immune cells constitutes a tightly coupled regulatory circuit that integrates bone and immune homeostasis. Activation of M1 macrophages and Th17 cells promotes RANKL-mediated osteoclastogenesis and resorptive activity, while impaired M2 and Treg signaling diminishes anti-inflammatory regulation and osteogenic repair [118,121]. This imbalance creates a sustained inflammatory state that weakens matrix stability, impairs angiogenic support, and perpetuates bone loss, mirroring the osteoimmune dysregulation observed in ONFH [122].

Together, these findings highlight osteoclast heterogeneity as key mediators of the cross-talk between immunity and bone remodeling. Their capacity to integrate inflammatory signals and translate them into cellular responses places them at the core of microenvironmental regulation. Disruption of this fine-tuned network provides a mechanistic link between chronic inflammation and failed bone regeneration, underscoring osteoclast heterogeneity as a potential therapeutic target for reestablishing immune and structural homeostasis in ONFH.

In summary, osteoclast heterogeneity plays an essential role in maintaining bone microenvironmental homeostasis through the coordinated regulation of bone remodeling, angiogenesis, and osteoimmunity. Disruption of this equilibrium directly links osteoclast functional imbalance to ONFH pathogenesis. This imbalance triggers a self-perpetuating cycle in which ischemia, oxidative stress, and inflammation collectively exacerbate microenvironmental disruption and structural deterioration, ultimately driving necrosis progression. These insights highlight osteoclast heterogeneity as the cellular nexus integrating vascular, osteogenic, and immune dysfunctions in ONFH.

Given their central regulatory role, osteoclasts have emerged as promising targets for bioactive scaffold design in ONFH therapy. While current strategies mainly suppress bone resorption to prevent structural collapse, few address the functional diversity of osteoclast populations or harness their reparative and pro-angiogenic potential. Incorporating osteoclast heterogeneity into scaffold design could enable dynamic reprogramming of the necrotic microenvironment, achieving synchronized control of osteogenesis, angiogenesis, and osteoimmunity for functional bone regeneration.

Recent studies have identified 2 functional osteoclast populations within the bone microenvironment: vessel-associated osteoclasts (VAOs) and bone-associated osteoclasts (BAOs) [87,123,124]. VAOs facilitate H-type vessel formation and osteogenesis, contributing to angiogenic–osteogenic coupling and microenvironmental stability, whereas BAOs primarily mediate bone matrix resorption and remodeling. Imbalance between these subsets disrupts osteogenic–vascular homeostasis, leading to impaired bone renewal and vascular regression [87]. Building on this framework, our recent work demonstrated that restoring VAO–BAO balance using GPC@miR reestablishes coordinated osteogenic and angiogenic activity, thereby promoting femoral head repair in ONFH [87]. These findings highlight the therapeutic potential of targeting osteoclast heterogeneity dynamics to restore coupled angiogenic and osteogenic remodeling.

Despite these promising insights, current research on osteoclast heterogeneity still faces notable methodological and translational limitations. A major challenge lies in identifying stable and specific molecular markers that can reliably distinguish distinct osteoclast populations under different physiological and pathological conditions. The lack of such markers complicates in vivo tracking and functional validation. Moreover, real-time lineage tracing and intravital imaging techniques for osteoclasts remain technically demanding, limiting the ability to monitor population dynamics during bone remodeling or necrosis progression. In addition, most mechanistic findings are derived from rodent models or in vitro systems, whereas single-cell and spatial transcriptomic analyses of human ONFH samples are still scarce, restricting direct clinical translation. From a therapeutic perspective, bioactive scaffolds designed to modulate osteoclast function may also cause nonspecific signaling effects due to overlapping pathways with other cell types. Addressing these limitations will require integrated approaches combining advanced imaging, multiomics profiling, and controlled in vivo models to establish a more precise and clinically translatable understanding of osteoclast heterogeneity in ONFH. Such progress will lay the groundwork for developing targeted strategies to restore bone homeostasis in ONFH and related osteolytic conditions.

## Summary and Outlook

ONFH, characterized by complex pathogenesis and rapid progression toward structural collapse, remains a major clinical challenge. This review has summarized current insights into ONFH pathophysiology, with particular emphasis on bone microenvironmental dysregulation. Core pathological features include imbalanced bone remodeling, impaired angiogenic function, and disrupted osteoimmune regulation. Recent advances in bioactive scaffolds have introduced promising therapeutic strategies that actively address these dysfunctions. By integrating bioactive components such as small molecules, growth factors, stem cells, exosomes, gene vectors, and metal ions, these materials restore microenvironmental balance and promote vascular regeneration, bone formation, and immune modulation. These developments mark a transition from passive structural support to dynamic regulation of the local tissue environment.

Growing recognition of osteoclast heterogeneity has further opened new avenues for therapeutic design. Functionally diverse osteoclast populations regulate not only bone resorption but also angiogenesis, osteogenesis, and osteoimmune responses, making them critical targets for scaffold-based strategies. Bioactive scaffolds that inhibit excessive resorption, enhance angiogenic–osteogenic coupling, or modulate osteoimmune activity by fine-tuning osteoclast functional diversity can help reestablish microenvironmental balance and mitigate disease progression.

Despite encouraging progress, clinical translation remains limited. Most bioactive scaffolds are still in preclinical stages, facing obstacles such as limited long-term biosafety data, scalability concerns, and incomplete understanding of bioactive component interactions within the human bone microenvironment. Scaffold designs that specifically leverage osteoclast heterogeneity are particularly nascent. To move forward, future studies must employ advanced techniques such as single-cell sequencing and spatial transcriptomics to define the roles and regulatory networks of osteoclast populations under both physiological and pathological conditions. These insights will support the rational development of bioactive scaffolds that fine-tune osteoclast function and promote tissue regeneration.

Further innovation should focus on creating responsive delivery systems that react to microenvironmental cues such as pH variation, oxidative stress, or inflammatory cytokines. Such systems would enable spatially and temporally controlled release of therapeutic agents, aligning treatment with disease dynamics and achieving targeted regulation of angiogenesis, osteogenesis, and immune activity. In addition, multifunctional bioactive scaffolds that combine vascular, osteogenic, and immunomodulatory properties will be essential for addressing the complex pathology of ONFH. The integration of environmental responsiveness with synergistic therapeutic effects will be key to advancing from laboratory research to clinical application.

Bridging the gap between fundamental discovery and clinical application requires not only technological innovation but also genuine cross-disciplinary integration. Mechanistic insights into osteoclast heterogeneity and bone microenvironmental dynamics should directly inform scaffold engineering by defining biological targets, guiding the choice of responsive materials, and tuning release kinetics to align with staged tissue remodeling. From an engineering perspective, reproducible fabrication, mechanical robustness under physiological loading, and scalable quality control are prerequisites for clinical readiness. In parallel, surgical feasibility demands that scaffold architecture and delivery formats integrate with established procedures such as core decompression and grafting, enabling minimally invasive and patient-specific implantation. True translational progress will rely on an iterative engineering–surgical codesign process in which feedback from clinical practice continuously refines material formulations and functional performance. Establishing such an interdisciplinary feedback loop can transform osteoclast-targeted bioactive scaffolds from conceptual constructs into realistic, clinically deployable interventions.

In conclusion, the convergence of biomaterial engineering and osteoclast biology provides a promising framework for the treatment of ONFH. By combining mechanistic understanding of cellular regulation with precision material design, this interdisciplinary approach offers the potential to shift treatment strategies from symptom management to true regeneration, bringing renewed hope to patients affected by this debilitating disease.
